# Minute-Ventilation Variability during Cardiopulmonary Exercise Test is Higher in Sedentary Men Than in Athletes

**DOI:** 10.5935/abc.20170104

**Published:** 2017-09

**Authors:** Renata Rodrigues Teixeira de Castro, Sabrina Pedrosa Lima, Allan Robson Kluser Sales, Antonio Claudio Lucas da Nóbrega

**Affiliations:** 1 Laboratório de Ciências do Exercício (LACE) - Universidade Federal Fluminense (UFF), Niterói, RJ - Brazil; 2 Hospital Naval Marcílio Dias - Marinha do Brazil, Rio de Janeiro, RJ - Brazil

**Keywords:** Breathing, Exercise, Respiratory Function Tests, Sedentary Lifestyle, Athletes, Pulmonary Ventilation, Exercise

## Abstract

**Background:**

The occurrence of minute-ventilation oscillations during exercise, named periodic breathing, exhibits important prognostic information in heart failure. Considering that exercise training could influence the fluctuation of ventilatory components during exercise, we hypothesized that ventilatory variability during exercise would be greater in sedentary men than athletes.

**Objective:**

To compare time-domain variability of ventilatory components of sedentary healthy men and athletes during a progressive maximal exercise test, evaluating their relationship to other variables usually obtained during a cardiopulmonary exercise test.

**Methods:**

Analysis of time-domain variability (SD/n and RMSSD/n) of minute-ventilation (Ve), respiratory rate (RR) and tidal volume (Vt) during a maximal cardiopulmonary exercise test of 9 athletes and 9 sedentary men was performed. Data was compared by two-tailed Student T test and Pearson´s correlations test.

**Results:**

Sedentary men exhibited greater Vt (SD/n: 1.6 ± 0.3 vs. 0.9 ± 0.3 mL/breaths; p < 0.001) and Ve (SD/n: 97.5 ± 23.1 vs. 71.6 ± 4.8 mL/min x breaths; p = 0.038) variabilities than athletes. VE/VCO_2_ correlated to Vt variability (RMSSD/n) in both groups.

**Conclusions:**

Time-domain variability of Vt and Ve during exercise is greater in sedentary than athletes, with a positive relationship between VE/VCO_2_ pointing to a possible influence of ventilation-perfusion ratio on ventilatory variability during exercise in healthy volunteers.

## Introduction

During a progressively increasing work rate exercise test, ventilation is expected to exhibit a curvilinear behavior when plotted against time, as work rate is increased above anaerobic threshold.^1^ Some heart failure patients' ventilation versus time plot does not comply with this physiological pattern and exhibits oscillations, with sequenced ups and downs in their ventilation versus time graphics during a cardiopulmonary exercise test. The presence of abnormal ventilatory oscillations in exercise test, named periodic breathing, is a powerful predictor of adverse outcome which prevalence varies from 25 to 31% of heart failure patients, depending on the criteria used to define it^2^ and regardless of the presence of other classic prognostic parameters.^3,4^

Recently, the prognostic value of oscillatory ventilation has been described in patients with heart failure with preserved ejection fraction^5,6^ and its occurrence has been described in apparently healthy people.^7^ Despite the prognostic value of this ventilatory parameter, there is still disagreement about the criteria that should be used to detect this phenomenon.^2,8^ Noteworthy, many variables that indicate prognosis in cardiopulmonary exercise tests are analyzed in a dichotomized approach. This means that a cut-off point categorize patients regarding their risk. Although this is convenient, there may be loss of important information.^9^ In fact, we have previously shown that some patients' ventilation versus time plot exhibits modest oscillations that although are not normal neither comply to any established criteria of periodic breathing.^10^ Thus there is a grey area of ventilation variability pattern that is usually neglected by a binary approach. This is probably indicating that periodic breathing is the abnormal extreme of a more insidious process characterized by the inability to keep minute ventilation varying around an accepted set point. Thus, a method capable of quantifying the ventilation variability may not only add to the understanding of ventilatory patterns during exercise, but also to analyze prognosis in a leveled approach that could be more detailed than a binary one.

Time-domain variability techniques are used in cardiology for the analysis of heart rate variability. We have previously replicated this technique to analyze ventilatory variability in heart failure patients during a maximal exercise test.^10,11^

Exercise training confers adaptations capable of modifying not only resting ventilatory parameters, but also their acute responses to a single exercise section.^12^ The adaptability of ventilatory variability to physical training is still unknown, but we have previously reported the reversal of periodic breathing, and reduction of ventilatory variability, after 14 weeks of cardiac rehabilitation in a patient with heart failure.^10^

Considering that exercise training could influence the fluctuation of ventilation during a progressive exercise test, we hypothesized that time-domain ventilatory variability during exercise would be greater in sedentary men than in athletes. Thus, the present study was designed to compare time-domain minute-ventilation variability of sedentary healthy men and athletes during a progressive maximal exercise test.

## Methods

### Volunteers

Eighteen male volunteers (9 sedentary and 9 athletes) were invited to participate in the study. All of them were considered healthy after clinical history and physical examination. None of them was a smoker or had been in regular use of any medication. Sedentary men were not involved in any regular physical activity during the last three months and have never been considered as athletes before. Athletes were professional soccer players from the same soccer team, playing first division in Rio de Janeiro, Brazil.

### Study protocol

All volunteers provided written informed consent to participate in the study after full explanation of the procedures and their potential risks. The investigation conformed to the principles outlined in the Declaration of Helsinki and have been approved by the Institutional Research Ethics Committee on Human Research.

All volunteers performed a maximal cardiopulmonary treadmill (Trackmaster 30x30, USA) exercise test following an individualized ramp protocol up to exhaustion. All tests achieved at least three of the following criteria to be considered maximum:^13^ achievement of oxygen consumption (VO_2_) plateau; perceived exertion (modified BORG scale) = 10; achievement of maximal predicted heart rate (220-age); respiratory exchange ratio ≥ 1,10.

Cardiopulmonary exercise tests were performed with gas exchange and ventilatory variables being analyzed breath-by-breath using a calibrated computer-based exercise system (*Ultima CardiO_2_ System*, Medical Graphics Corporation, USA). The O_2_ and CO_2_ analyzers were calibrated before each test using a reference gas (12% O_2_; 5% CO_2_; nitrogen balance). The pneumotachograph used was also calibrated, with a 3L syringe using different flow profiles. During each cardiopulmonary exercise test, a 12-lead electrocardiogram was continuously recorded (*Cardioperfect*, Welch Allin, USA) and heart rate automatically derived. Carbon dioxide production (CO_2_), VO_2_, tidal volume (Vt) and respiratory rate (RR), were registered breath-by-breath. Minute ventilation (VE), O_2_ and CO_2_ ventilatory equivalents (VE/VO_2_ and VE/VCO_2_) were automatically calculated (*Breeze Software* 6.4.1, Medical Graphics, USA). All breath-by-breath results were exported to an Excel spreadsheet (*Microsoft Corporation*, USA), where standard deviation (SD) and root mean square successive difference (RMSSD) of VE during exercise test were calculated for each patient. Considering that the number of observations has a direct influence on variability measurement, results (SD and RMSSD) were normalized to the number of respiratory cycles during the test, reducing the probability that a greater number of observations registered in longer tests would be the sole responsible for greater variability (SD/n and RMSSD/n, respectively).^14^

### Statistical analysis

Statistical analysis was performed using the software Statistica 7.0 (*Statsoft Inc,* USA). Variables from the cardiopulmonary exercise tests showed normal distribution when analyzed by the *Shapiro Wilk*'s test. Exercise variables in both groups were compared by paired two-tailed Student T test. Significance was set at p < 0.05. Results are presented as mean ± standard deviation.

A sample size of twelve individuals (6 in each group) would be needed to provide an 80% power with a 2-sided alpha of 0.05 to detect a difference of 10 ± 5 ml/min x breaths in SD/n ventilation variability between the two groups. Considering that ventilatory variability is a new variable, and that there are no published data to guide us regarding expected values, we have decided to increase sample in 50% and that is why the presented study included 18 individuals. After finishing the study, the calculated power of ventilatory variability is 100%.

## Results

The demographic and anthropometric characteristics of both groups are described in table 1. All tests achieved the oxygen consumption plateau and a respiratory quotient greater than 1.10, and thus were considered maximum testes. Peak cardiopulmonary exercise data of both groups are shown in table 2.

**Table 1:** Demographic and anthropometric data of volunteers (n = 18)

Variable	Sedentary men (n = 9)			Athletes (n = 9)			p value*
Age (years)	26	±	6	22	±	2	0.128
Weight (kg)	77.7	±	11.0	70.6	±	1.3	0.134
Height (m)	1.75	±	0.06	1.75	±	0.03	0.866
BMI (kg/m^2^)	25.4	±	3.04	23.05	±	1.14	0.064

* Comparison between groups by student T test. BMI: body mass index.

**Table 2:** Peak exercise data during graded maximal cardiopulmonary exercise test performed by athletes and sedentary men in a treadmill

	Athletes (n = 9)			Sedentary men (n = 9)			p value
VO_2_ (mL/kg/min)	47.8	±	0.3	42.6	±	4.2	0.029
VCO_2_ (mL/kg/min)	64.1	±	1.2	54.8	±	6.0	0.009
RER	1.3	±	0.3	1.29	±	0.3	0.380
Ve (L/min)	128.7	±	0.3	123.4	±	14.7	0.550
Respiratory rate (breaths/min)	57	±	3	54	±	6	0.540
Vt (L)	2.3	±	0.3	2.3	±	0.3	0.837
Heart rate (beats/min)	181	±	3	186	±	3	0.343
VE/VO_2_	2.7	±	0.3	2.9	±	0.3	0.309
VE/VCO_2_	2.0	±	0.3	2.3	±	0.3	0.106
RR/VO_2_(breaths/ml/Kg/min)	1.2	±	0.3	1.3	±	0.3	0.363
VO_2_/HR (ml/ beat)	0.3	±	0.3	0.2	±	0.3	0.015

VO_2_: peak oxygen consumption; VCO_2_: peak carbon dioxide production; RER: respiratory exchange ratio; Ve: minute-ventilation; Vt: tidal volume. P value refers to the result of paired student’s T test.

**Figure 1:**
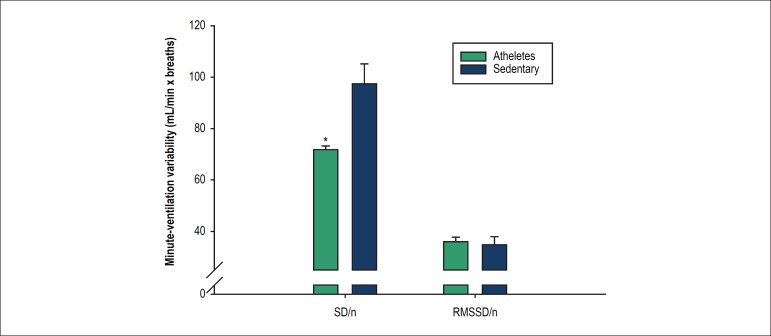
Minute-ventilation variability (SD/n and RMSSD/n) of athletes (green bars) and sedentary men (blue bars) during a graded maximal exercise test. * p < 0.05 vs. sedentary.

Sedentary men exhibited higher time-domain variability of minute-ventilation than athletes during cardiopulmonary exercise test, as showed in figure 1.

## Discussion

The analysis of minute-ventilation curve during exercise has gained interest since the first reports of exercise oscillatory ventilation.^15,16^ Although a lot of progress has been done regarding the prognosis value of this phenomenon since then,^4,6,17^ there was almost no progress in the quantification of this phenomenon.^18^ There are currently two major diagnostic definitions of exercise oscillatory ventilation.^3,17^ Both definitions require the visualization of the ventilatory pattern during exercise to determine the presence or absence of exercise oscillatory ventilation, in a dichotomized way. We have previously shown that applying time-domain variability techniques can be easily performed and may help quantifying exercise ventilatory oscillations. Olson and Johnson^18^ have also proposed a software application to quantify measures of exercise oscillatory ventilation in heart failure patients.

Considering that most ventilatory parameters exhibit some adaptation to physical training, it is conceivable to hypothesize that ventilatory variability would also be affected by chronic exposure to physical exercise. The present study compared ventilatory variability throughout exercise in athletes and sedentary men and concluded that untrained volunteers exhibited greater minute-ventilation variability than soccer athletes.

It is important to note that all volunteers were healthy and without any cardiovascular or respiratory disease. Therefore, some mechanisms involved in *Cheyne-Stokes* respiration and periodic breathing, such as hypocapnia, and pulmonary blood flow fluctuations,^19^ which are considered key mechanisms of periodic breathing in heart failure, would probably not be useful in understanding physiological ventilatory variability during exercise in healthy subjects. Increased central and peripheral chemosensitivity^20^ is also involved with *Cheyne-Stokes* respiration. Ohyabu et al showed that ventilatory sensitivity during hypoxia was attenuated in long-distance runners and sprinters compared to non-athletes.^21^ In fact, endurance training reduces the ventilatory response to a given level of work due to an attenuated chemosensitivity.^22,23^ So, it is possible that reduced chemosensitivity would explain the findings of the present study.

Although there was no statistical difference regarding weight or body mass index between groups, volunteers in the sedentary group exhibited near significantly higher weight and BMI. One could hypothesize that this slight and non-significant difference could have influenced the different breathing patterns found in the study. In fact, in morbid obese individuals, excessive body weight can induce chest wall restriction^24^ and loosing body weight may improve lung function.^25^ Nevertheless, although there was some overweight volunteers in both groups, there was not a single obese volunteer in this study. We could not found any study that compared ventilatory parameters in overweight and not overweight individuals during exercise. Regarding rest breathing patterns, it seems that body mass only influences lung function when obese individuals are in supine position. Our volunteers were non-obese and all tests were performed in the upright position. Thus, it seems unlikely that the slight and non significant difference in BMI between groups would have influenced the ventilatory variability results of the present study.

The analysis of table 2 shows maximal VO_2_ that is not as high as expected for professional soccer players. There are several possible explanations for this finding. First of all, data was collected in the beginning of the season, just after holydays. So, athletes were not in their best shape. It is also important to note that there is clear VO_2max_ variation profiles between soccer players accordingly to their playing position and style.^26^ We have included athletes from all playing positions, from the same team, in the athlete group. So, there were differences between their VO_2max_. Finally, players in Brazil appear to be shorter in stature, similar in body mass and have a lower overall aerobic capacity when compared to their European equivalents.^26^

### Study limitations

Some operational and technical aspects could have influenced the results of the present study. Subjects were not submitted to rest pulmonary function tests before entering the study. Considering none of them had any past history of pulmonary disease or smoking, the absence of rest pulmonary function tests, although desirable, does not seem to be a major issue influencing the present results.

The use of different interfaces to breath analysis may influence the depth and rate of breathing.^27^ Although this effect appears to be restricted to lower levels of exercise,^28^ it seems reasonable not to interchangeably compare ventilatory variability results recorded using mask, mouthpiece or canopy. All breath-by-breath data in this study was collected throughout a face mask. Thus, the selected interface could not have influenced the different results when both groups were compared.

This is a cross-sectional study where trained and untrained men were compared. A study that evaluates the effects of physical training would rather have a longitudinal than the present design. Nevertheless, the only difference between both studied groups was their peak VO_2_, which was higher in athletes, as expected. Thus, although athletes were not longitudinally evaluated, it seems that the different exercise responses in both groups could be directly attributable to physical training.

## Conclusions

The presence of periodic breathing is a powerful predictor of adverse outcome in heart failure.^2^ This is an extreme presentation of a ventilatory variation that although unseen by our eyes can be mathematically calculated. The present study adds information regarding the quantification of exercise minute-ventilation variability, and points to the direction that this is a trainable exercise variable. The exact mechanisms that influence ventilatory variability during exercise remain to be studied.
